# Dynamic ^31^P–MRSI using spiral spectroscopic imaging can map mitochondrial capacity in muscles of the human calf during plantar flexion exercise at 7 T

**DOI:** 10.1002/nbm.3662

**Published:** 2016-11-08

**Authors:** Ladislav Valkovič, Marek Chmelík, Martin Meyerspeer, Borjan Gagoski, Christopher T. Rodgers, Martin Krššák, Ovidiu C. Andronesi, Siegfried Trattnig, Wolfgang Bogner

**Affiliations:** ^1^ High‐Field MR CentreMedical University of ViennaViennaAustria; ^2^ Department of Biomedical Imaging and Image‐Guided TherapyMedical University of ViennaViennaAustria; ^3^ Christian Doppler Laboratory for Clinical Molecular MR ImagingViennaAustria; ^4^ Department of Imaging Methods, Institute of Measurement ScienceSlovak Academy of SciencesBratislavaSlovakia; ^5^ Oxford Centre for Clinical Magnetic Resonance Research (OCMR)University of OxfordOxfordUK; ^6^ Center for Medical Physics and Biomedical EngineeringMedical University of ViennaViennaAustria; ^7^ Fetal Neonatal Neuroimaging and Developmental Science CenterBoston Children's HospitalBostonMassachusettsUSA; ^8^ Division of Endocrinology and Metabolism, Department of Internal Medicine IIIMedical University of ViennaViennaAustria; ^9^ Athinoula A. Martinos Center for Biomedical Imaging, Department of Radiology, Massachusetts General HospitalHarvard Medical SchoolBostonMassachusettsUSA

**Keywords:** dynamic ^31^P–MRS, high energy phosphate, MRSI, skeletal muscle, spiral spectroscopic imaging, ultra‐high field

## Abstract

Phosphorus MRSI (^31^P–MRSI) using a spiral‐trajectory readout at 7 T was developed for high temporal resolution mapping of the mitochondrial capacity of exercising human skeletal muscle.

The sensitivity and localization accuracy of the method was investigated in phantoms. *In vivo* performance was assessed in 12 volunteers, who performed a plantar flexion exercise inside a whole‐body 7 T MR scanner using an MR‐compatible ergometer and a surface coil. In five volunteers the knee was flexed (~60°) to shift the major workload from the gastrocnemii to the soleus muscle.

Spiral‐encoded MRSI provided 16–25 times faster mapping with a better point spread function than elliptical phase‐encoded MRSI with the same matrix size. The inevitable trade‐off for the increased temporal resolution was a reduced signal‐to‐noise ratio, but this was acceptable. The phosphocreatine (PCr) depletion caused by exercise at 0° knee angulation was significantly higher in both gastrocnemii than in the soleus (i.e. 64.8 ± 19.6% and 65.9 ± 23.6% in gastrocnemius lateralis and medialis versus 15.3 ± 8.4% in the soleus).

Spiral‐encoded ^31^P–MRSI is a powerful tool for dynamic mapping of exercising muscle oxidative metabolism, including localized assessment of PCr concentrations, pH and maximal oxidative flux with high temporal and spatial resolution.

**Abbreviations used**
^31^P–MRI: phosphorus MRI
^31^P–MRS: phosphorus MRS
^31^P–MRSI: phosphorus MRSI
ADP: adenosine diphosphate
ATP: adenosine triphosphate
FFT: fast Fourier transform
FOV: field of view
FWHM: full width at half maximum
GL: gastrocnemius lateralis
GM: gastrocnemius medialis
GPC: glycerophosphocholine
NA: number of averages
PAD: peripheral arterial disease
PCr: phosphocreatine
PSF: point spread function
SAR: specific absorption rate
SNR: signal‐to‐noise ratio
SOL: soleus
SVS: single‐voxel spectroscopy
*T*_E_*: acquisition delay

## 1 INTRODUCTION

Impaired energy metabolism in skeletal muscle mitochondria is indicative of muscular disorders (e.g. Duchenne muscular dystrophy^1^ or mitochondrial myopathy^2^), systemic metabolic diseases (e.g. diabetes mellitus^3^, ^4^) and cardiovascular diseases (e.g. peripheral arterial disease (PAD)^5^, ^6^, ^7^). These observations, together with the fact that evaluation of muscle oxidative metabolism provides insight into personal training status,^8^, ^9^ have led to a considerable methodological development in medical imaging for non‐invasive assessment of muscle energy metabolism.

In particular, phosphorus MRS (^31^P–MRS) has been established as a powerful tool for studies of energy metabolism.^10^, ^11^, ^12^, ^13^, ^14^, ^15^ More specifically, dynamic ^31^P–MRS, during exercise and recovery, allows direct estimation of the oxidative adenosine triphosphate (ATP) synthesis rate in challenged muscle, which reflects maximal mitochondrial capacity.^12^ Since high temporal resolution (of the order of seconds) is required to map the phosphocreatine (PCr) recovery curve, coarse signal localization restricted only by the sensitive volume of a surface coil is often used to retain sufficient signal‐to‐noise ratio (SNR) for dynamic ^31^P–MRS experiments.^16^, ^17^ Such localization cannot distinguish between muscle groups that are recruited differently in the performed exercise (e.g. soleus (SOL) and gastrocnemius during plantar flexion^18^, ^19^, ^20^, ^21^, ^22^, ^23^).

Single‐voxel MRS^24^, ^25^ or slab‐selective MRS^26^, ^27^ have been implemented to overcome this limitation and enable single‐muscle localization. However, spatially defined injuries, myopathies, or functional deficits in PAD can be overlooked if only a single predefined muscle location is examined. Thus, actual mapping approaches allowing for coverage of several muscle groups simultaneously are desirable. Frequency‐selective phosphorus MRI (^31^P–MRI) has been shown to provide information on PCr recovery kinetics from several muscles simultaneously,^28^, ^29^, ^30^ and was recently extended to provide information about pH dynamics during exercise.^31^ So far, these ^31^P–MRI techniques do not provide the same amount of information as ^31^P–MRS; e.g., there are no data on ATP signal intensity typically used for concentration quantification, or on other ^31^P metabolites. The temporal resolution of phosphorus MRSI (^31^P–MRSI) with Cartesian phase encoding is, however, insufficient for estimating mitochondrial capacity with high spatial resolution unless a dedicated exercise protocol (i.e. gated MRSI^32^, ^33^) is applied, which considerably complicates and prolongs the examination and may impose limitations, such as the requirement of only mild pH changes.

In this context, our aim was to develop and test a ^31^P–MRSI sequence using spiral readout trajectories with high temporal resolution for spatially resolved quantification of maximal oxidative ATP‐synthase flux in the muscles of the human calf during plantar flexion exercise. The performance of the proposed sequence was tested in a localization phantom and in healthy volunteers.

## 2 METHODS

### 2.1 Hardware

All MR measurements were performed on a 7 T MR system (Magnetom, Siemens Healthcare, Erlangen, Germany) using a 10 cm circular, dual‐tuned (^31^P/^1^H) surface‐coil (Rapid Biomedical, Rimpar, Germany). The MR system was equipped with an SC72 gradient system featuring 70 mT/m nominal gradient strength and 200 mT/m/s maximum gradient slew rate. For the dynamic *in vivo* experiments a dedicated plantar flexion ergometer (Trispect, Ergospect, Innsbruck, Austria) was used.

### 2.2 Sequence design

**Figure 1:**
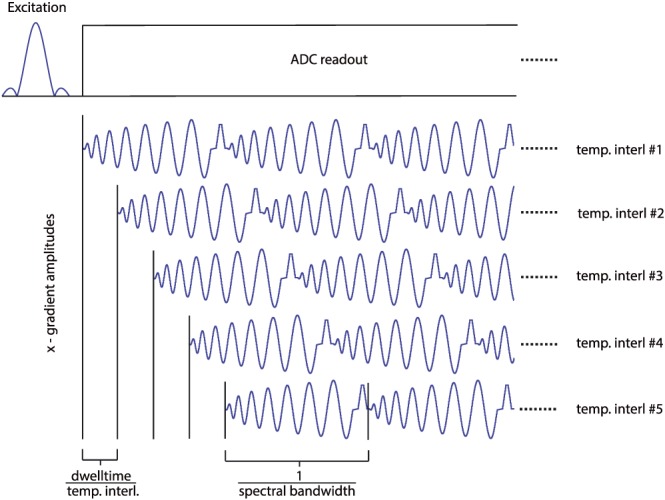
In‐plane constant density spiral readout for FOV 200 × 200 mm^2^ and 14 × 14 matrix with temporal interleaving. The slice‐selective excitation pulse is followed by the spiral gradient modulations (only *x* ‐direction shown). These spiral trajectories are played out repeatedly (512 times; only three shown for illustration) to cover the whole free induction decay in the time domain. As the duration of a spiral is too long to allow a sufficiently short spectral dwell time, temporal interleaves (five temporal interleaves are shown here for illustration) were acquired. Each temporal interleave is acquired after a separate excitation, and identical spiral gradients are played out with a predefined delay. This delay determines the actual dwell time and hence the spectral readout bandwidth. The number of temporal interleaves used in the sequence was derived as the minimum number required to cover the desired spectral bandwidth of 1.4 kHz

To accelerate the acquisition and hence increase the temporal resolution of our dynamic ^31^P–MRSI scans, we implemented a constant‐density spiral spectroscopic readout.^34^, ^35^ Following each slice‐selective excitation (achieved by a Hamming filtered SINC pulse with 600 μs duration and 6.4 kHz bandwidth), we played out consecutive and identical spiral gradient waveforms (*n* = 512 divided by the number of temporal interleaves) in the read (*x*) and phase (*y*) gradient directions that fully covered the *k*‐*t* space. The spiral trajectories for all excitations were identical. Since the duration of the spiral trajectories for the given voxel size was longer than the temporal sampling required for the desired spectral bandwidth, temporal interleaving (i.e. shifting the gradient trajectory for consecutive *T*_R_ by a fraction of the dwell time) was employed (Figure 1) (e.g., for five temporal interleaves at 1.45 kHz spectral bandwidth 103 spirals of about 3.4 ms length per spiral were played out per interleave, leading to a total sampling duration of about 353 ms, with each interleave being delayed by 0.69 ms relative to its predecessor). To optimize the SNR, gradient rewinders (i.e. deadtime during readout that is necessary to return to the *k*‐space center) were minimized (<6%). Hence, only temporal interleaves were used and not spatial interleaves. The number of temporal interleaves was adjusted to the targeted matrix size, making sure that the spectral bandwidth remained between 1.4 and 1.45 kHz (i.e. covering a range of at least 12 ppm from P_i_ to γ‐ATP resonances). The achieved spectral resolution, i.e. the spectral bandwidth over the number of acquired spirals, was therefore always 2.7–2.8 Hz. The entire spiral trajectory calculation and data reconstruction were implemented on the MR scanner. The following four steps were performed within the online processing pipeline: (i) gradient delays between the ADC readout and the *x*/*y* gradients of 8 μs were corrected by shifting the sampled data inside the array; (ii) the acquired data were gridded two‐dimensionally to a twofold oversampled Cartesian matrix with a Kaiser–Bessel kernel, (iii) a fast Fourier transform (FFT) was applied in the *x*/*y* dimension, and then (iv) an FFT was used in the frequency dimension.

### 2.3 Phantom experiments

The point spread function (PSF) of the proposed MRSI sequence was measured in a two‐compartment phantom. The outer compartment (a cylinder with a diameter of 13 cm) was filled with tap water, and the inner compartment (a cube with 9 mm inner side length) was filled with a P_i_ solution (concentration = 1 mol/L). The cube was fixed in the center of the cylinder, 3 cm from its base. Three 2D–MRSI datasets (slice thickness = 30 mm) were collected in the transverse plane, perpendicular to the RF coil and covering the inner P_i_‐filled compartment entirely, using (i) standard 2D–MRSI with fully sampled Cartesian *k*‐space (phase encoded), (ii) elliptically sampled Cartesian *k* space, and (iii) constant density spiral *k*‐space sampling. The acquisition parameters were matched for all three sequences: acquisition delay (*T*_E_*) = 1 ms; *T*_R_ = 2 s; nominal flip angle =90°; field of view (FOV) = 200 × 200 mm^2^; acquisition matrix size =14 × 14. In the first step, the number of averages (NA) was set to 1. The corresponding acquisition times were 6 min 32 s for full phase encoding, 3 min 46 s for elliptical phase encoding, and 10 s for spiral encoding. To compare the spatial selectivities of all sampling approaches, the full width at half maximum (FWHM) of the PSF was measured. Additionally, to compare the SNRs of the MRSI sequences with elliptical phase‐encoding and spiral encoding, the NA of the spiral‐encoded sequence was increased to match the acquisition time of the elliptical Cartesian encoding (i.e., NA = 22, acquisition time = 3 min 40 s).

### 2.4 *In vivo* measurements

To test the potential temporal and spatial resolution of the proposed sequence in an *in vivo* situation, one healthy male volunteer underwent repeated measurements of the calf muscle. The volunteer lay supine on the MR table and the RF coil was placed under the right gastrocnemius muscle. The matrix sizes used and corresponding numbers of required temporal interleaves are listed in Table 1. Other parameters were set as follows: *T*_E_* = 1 ms; *T*_R_ = 2 s; FOV = 200 × 200 mm^2^; slice thickness = 30 mm; vector size/total number of spirals for all temporal interleaves =512 points/spirals; NA = 1, 4, and 16. The SNR of PCr signal was calculated for each matrix size and NA.

**Table 1:** The relation between the matrix size (i.e. spatial resolution) and the number of temporal interleaves required (i.e. temporal resolution). For comparison, the temporal resolution of an elliptically phase‐encoded MRSI (ePE) is also given. Note that the increase in temporal resolution comes at the cost of SNR, which is, however, comparable once the acquisition time is matched and the differences in PSF between spiral and elliptical encoding are corrected for

Matrix size	Spatial resolution (mm^3^)	No of temporal interleaves	Temporal resolution (s)	
			Spirals	ePE
**10 × 10**	20 × 20 × 30	3	6	98
**12 × 12**	17 × 17 × 30	4	8	162
**14 × 14**	14 × 14 × 30	5	10	226
**16 × 16**	12 × 12 × 30	6	12	298

Twelve young, healthy individuals (nine males/three females, age (mean ± standard deviation) 28.7 ± 4.1 years, BMI (body mass index) 23.2 ± 2.6 kg/m^2^, no professional athletes) agreed to participate in the dynamic part of this study and signed a consent form approved by the institutional ethical board. Each volunteer lay supine on the ergometer, inside the MR system, with the RF coil strapped below their right calf. The knee of the volunteers was fully extended during the dynamic examination (2 min rest, 6 min exercise and 6 min recovery) to ensure a major involvement of the gastrocnemius muscles and only a minor contribution of the SOL muscle to the exercise performed.^20^, ^21^, ^22^ The volunteers performed plantar flexions at a workload set to about 25–35% of the maximal voluntary contraction force, once every *T*_R_ (2 s). The exercise was synchronized with the data acquisition based on an audio signal, so that the MRSI data were acquired when the calf muscle was relaxed. In addition, five of the recruited male volunteers underwent a second dynamic examination with the knee flexed at about 60°. In this leg position the SOL muscle is expected to be involved more than the two gastrocnemii.^20^ This second examination was performed at least 20 min after the first one, to ensure sufficient metabolic recovery.^36^ Two additional spiral‐MRSI measurements were performed at rest in two of these subjects in order to acquire representative flip angle maps^37^ in both leg positions.

For both dynamic examinations, the proposed acquisition sequence was used, with the following parameters: *T*_E_* = 1 ms; *T*_R_ = 2 s; nominal flip angle =52° (set about 2.5 cm deep from the coil, by recalculating the voltage that was required to obtain 90° excitation, i.e. maximum signal^38^); acquisition bandwidth =1450 Hz; vector size =512 points; four dummy scans. With matrix size =14 × 14, FOV = 200 × 200 mm^2^ and slice thickness = 30 mm, the nominal spatial resolution of the dynamic experiments was 14.3 × 14.3 × 30 mm^3^ (6.13 mL). The excitation frequency was centered at 180 Hz (~1.5 ppm) relative to PCr. The *T*_R_ values used for the flip angle map acquisition were 2 s and 8 s. Two averages were acquired and the rest of the sequence parameters were kept the same as for the dynamic experiments.

### 2.5 Data analysis

All acquired MRSI data were interpolated to 16 × 16 matrixes, and voxels with SNR of the PCr signal above 8 in the resting state before exercise (no *k*‐space filtering was applied prior to data analysis) were pre‐selected for quantification of dynamic *in vivo* data. Custom software written in IDL (Exelis Visual Information Solutions, Boulder, CO, USA) was used for pre‐selection of these voxels. All selected spectra were analyzed using the jMRUI software (Version 5.0) with the AMARES (advanced method for accurate, robust, and efficient spectral fitting) time domain fitting routine.^39^ The γ‐ATP resonance was used as an internal concentration reference, assuming a stable cellular ATP concentration of 8.2 mM.^40^ To improve the SNR of the γ‐ATP peak for quantification purposes, the γ‐ATP signal intensity was averaged over the last six measurements (1 min) of the recovery period. For the saturation correction, the actual flip angle was calculated for each muscle group as the average of the measured datasets, and the previously measured *T*_1_ values^41^ were used. The chemical shift of P_i_ relative to PCr was used to calculate the intracellular pH,^42^ according to the modified Henderson‐Hasselbalch equation.^40^

During the recovery period, a mono‐exponential function was fitted to the PCr signal time course using custom routine in MATLAB (MathWorks, Natick, MA, USA), yielding the time constant of the PCr recovery rate (*τ*_PCr_). The *τ*_PCr_ was used to calculate the initial PCr recovery rate (*V*_PCr_), as reported before.^15^ The maximal rate of oxidative phosphorylation (*Q*_max_) was calculated according to the adenosine diphosphate (ADP)‐based model of Michaelis and Menten, for which the ADP concentration was calculated according to Kemp et al.^43^

The SNR was calculated based on the magnitude of the signal from the voxel covering the cubic phantom for the phantom experiments, comparing the SNR of elliptical and spiral encodings matched for acquisition time, and similarly by using the magnitude of PCr signal in voxels within a 5 × 3 area covering the skeletal muscle tissue for the *in vivo* experiment that compared the SNR for different spatial resolutions. The standard deviation of noise evaluated from 100 points of the spectra far from the metabolite signals was used for SNR calculation.

To compare the oxidative metabolism between the three muscle groups of interest—gastrocnemius medialis (GM), gastrocnemius lateralis (GL) and SOL—the muscles were manually segmented using ^1^H localizer images. Each segmented volume of interest contained 3–5 voxels, leaving at least one voxel free between the adjoining muscles. One way analysis of variance (ANOVA) with a Tukey post hoc test was consecutively applied for statistical analysis, considering *p* < 0.05 statistically significant. A paired *t* test was used to compare the measures of mitochondrial function determined for the exercise with straightened (0°) and bent (60°) knee, with the same level of significance (*p* < 0.05).

## 3 RESULTS

**Figure 2:**
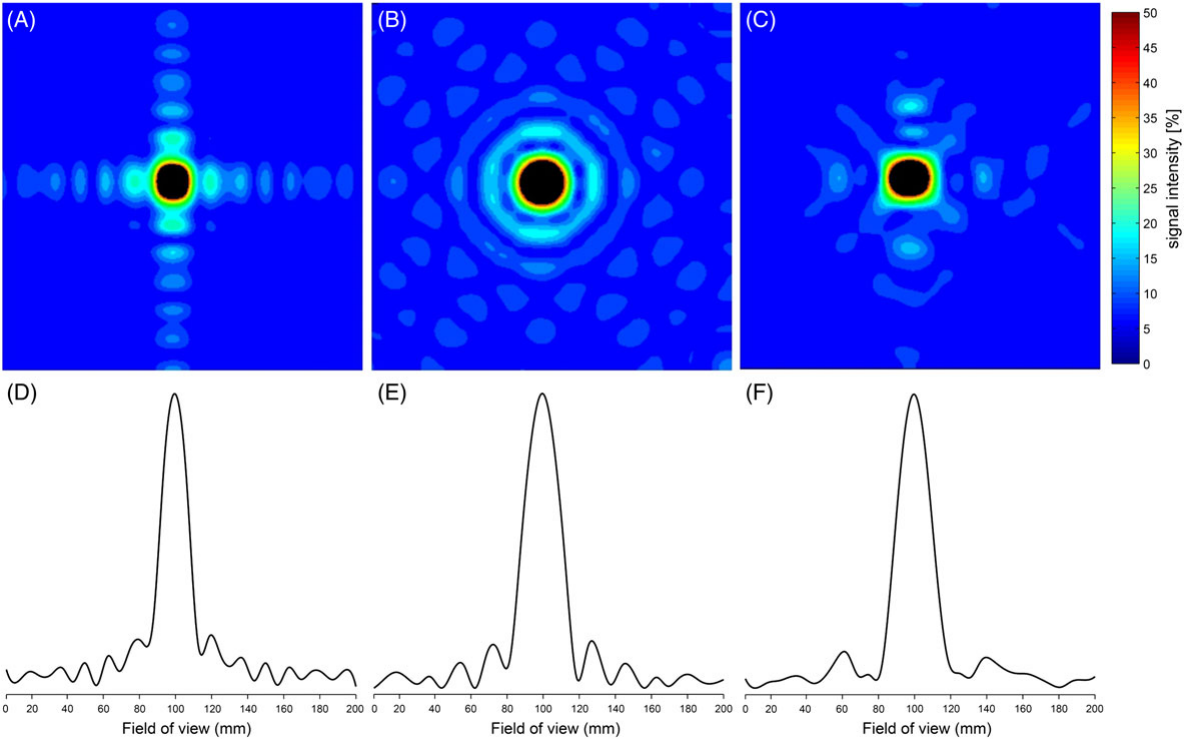
Experimentally determined PSFs for different *k*‐space sampling approaches for slice‐selective 2D–MRSI with a 14 × 14 matrix using a point source (i.e. inorganic phosphate solution) depicted as 2D maps (A‐C) and 1D plots (D‐F): A,D, full phase‐encoded *k*‐space sampling; B,E, elliptically phase‐encoded *k*‐space sampling; C,F, constant‐density spiral sampling with no spatial, but five temporal, interleaves. Constant‐density spirals featured a reduced number of side‐lobes in comparison with full phase encoding acquisition and provided smaller PSF than the elliptical encoding alternative (3.5 cm^2^ versus 4.9 cm^2^). All signal intensities are cut at 50% of the maximum signal intensity in the 2D maps to emphasize the FWHM. Note that all maps and plots are given in absolute values, i.e. all side‐lobes are depicted as positive

Spiral *k*‐space‐sampled MRSI provided 27% lower SNR than the elliptical‐encoded MRSI with the same acquisition time, i.e. 279.26 compared with 384.25 for spiral sampling (NA = 22) and elliptical phase encoding (NA = 1), respectively. However, the effective voxel size of the spiral‐encoded MRSI was 30% smaller, as the PSF area at FWHM was 3.5 cm^2^ versus 4.9 cm^2^ for the spiral and elliptical encoding, respectively. The PSF area at FWHM of the main lobe was 1.6 cm^2^ for the fully encoded *k*‐space. The results of the phantom PSF experiments are depicted in Figure 2.

The SNR comparison between different matrix sizes and temporal resolutions measured *in vivo* at rest is given in Table 2. Depending on the chosen matrix size, spiral MRSI encoding improved the minimum temporal resolution compared with elliptical phase‐encoded MRSI by a factor of 16 (10 × 10 matrix) or up to 25 (16 × 16 matrix). For more details see Table 1. A 14 × 14 matrix size was selected for the dynamic experiments, as a good compromise between spatial (effective voxel size 7.56 mL, i.e. cylinder with a base equal to 64% of the PSF area^44^ and height equal to the slice thickness) and temporal (10 s) resolution with sufficient SNR.

**Table 2:** The average SNR of PCr peak in a 5 × 3 area within the skeletal muscle for different matrix sizes for the spiral‐encoded MRSI

NA	10 × 10	12 × 12	14 × 14	16 × 16
1	26.9 ± 8.6	21.8 ± 6.7	14.7 ± 6.5	11.6 ± 4.8
4	50.8 ± 17.2	42.5 ± 13.3	26.8 ± 13.9	23.1 ± 10.3
16	101.4 ± 31.5	82.0 ± 27.7	52.9 ± 23.6	45.4 ± 20.9

**Figure 3:**
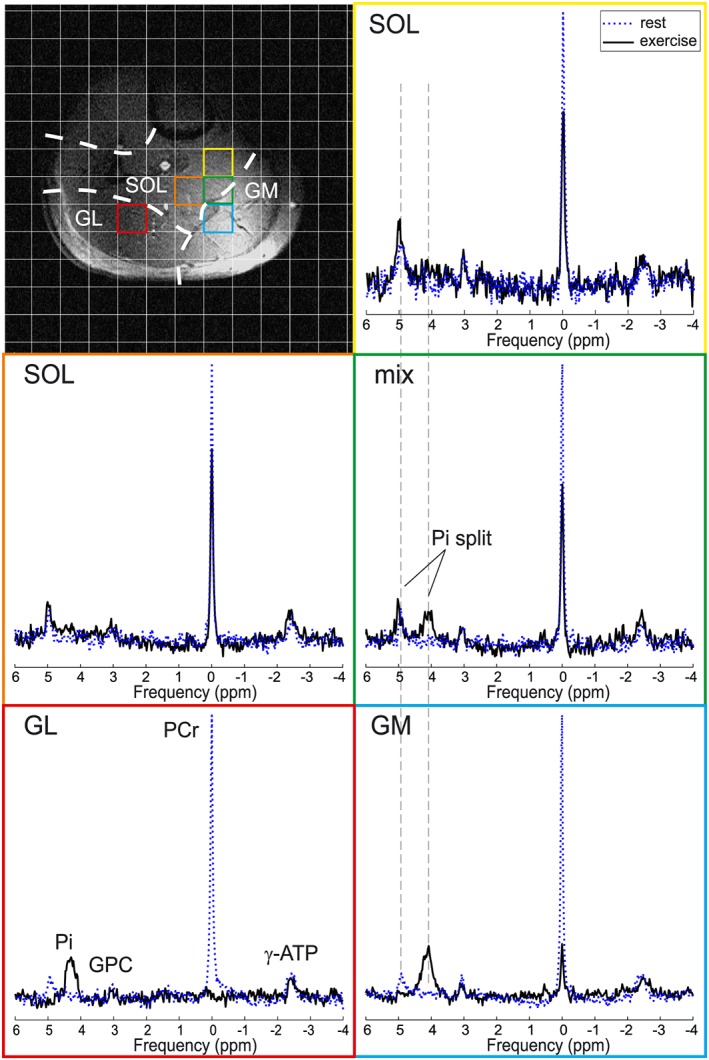
Representative ^31^P MR spectra acquired at rest (blue dotted line) and at the end of exercise (black solid line) in voxels representing single muscles, i.e. GL, GM and SOL, or a mixture of GM and SOL tissue. Note the P_i_ splitting in the mixed (green) voxel. A matched filter (6 Hz Lorentzian) was applied for visualization purposes only

Representative spectra acquired at rest and at the end of exercise from each investigated muscle group are depicted in Figure 3. To demonstrate the importance of ^31^P signal localization in dynamic experiments, a voxel containing a mixture of GM and SOL tissue is also visualized (green). Note that in such voxels of mixed tissue splitting of the P_i_ signal, which corresponds to two compartments with different pH values, can be observed.

**Figure 4:**
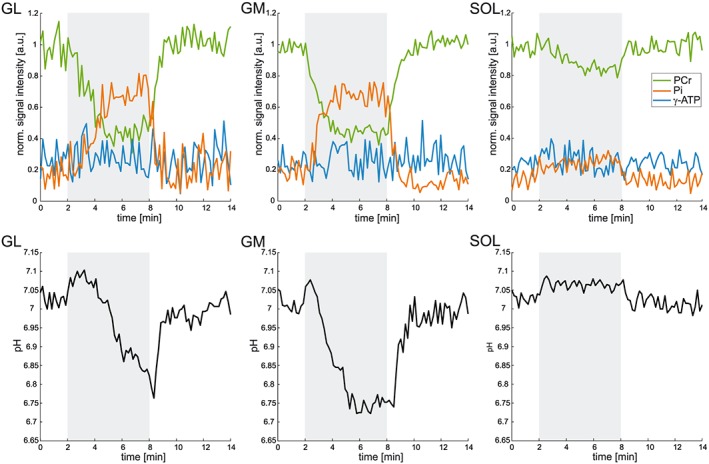
Time courses of the PCr (green), P_i_ (orange) and ATP (blue) signal intensities, normalized to the resting PCr signal intensity (top panel), and of pH (bottom panel), measured in all investigated muscles (2–4 voxels). The higher PCr depletion and pH drop in GL and GM indicate the prevailing involvement of these muscles in the plantar flexion exercise (0° flexion of the knee). The exercise period (6 min) is shaded grey

The time evolution of PCr and P_i_ signals—in GM, GL and SOL—during plantar flexion exercise with a straightened knee is depicted in Figure 4 (top panel). The time evolution of pH in these muscles is also included in Figure 4 (bottom panel). The mean PCr depletion of 15.3 ± 8.4% measured in the SOL muscle was significantly lower (*p* < 0.01) than that in GM and GL (65.9 ± 23.6% and 64.8 ± 19.6%, respectively). Similarly, significantly lower *V*_PCr_ and *Q*_max_ values were calculated for the SOL muscle in comparison with both gastrocnemii in this type of exercise. All measured and evaluated measures of mitochondrial function are listed in Table 3.

**Table 3:** ^31^P–MR measures of mitochondrial function measured and derived for GL, GM and SOL from the dynamic examination with straightened knee (*n* = 12)

	GL	GM	SOL
**PCr [mM]**	34.4 ± 4.2	37.0 ± 7.1	39.3 ± 5.4
**PCr drop [%]**	64.8 ± 19.6	65.9 ± 23.6	15.3 ± 8.4* ^,^ §
***τ***_**PCr**_ **[s]**	60.1 ± 21.4	63.1 ± 20.8	66.2 ± 16.0
**pH**_**end_exercise**_	6.79 ± 0.13	6.77 ± 0.18	6.93 ± 0.10#
***V***_**PCr**_ **[mM/s]**	0.41 ± 0.12	0.42 ± 0.16	0.10 ± 0.05* ^,^ §
***Q***_**max**_ **[mM/s]**	0.58 ± 0.17	0.57 ± 0.20	0.31 ± 0.10* ^,^ §

Data are given as mean ± standard deviation. Significant differences between muscle groups are depicted as follows:

* GL versus SOL (*p* < 0.01),

§ GM versus SOL (*p* < 0.01) and

# GM versus SOL (*p* < 0.05).

**Figure 5:**
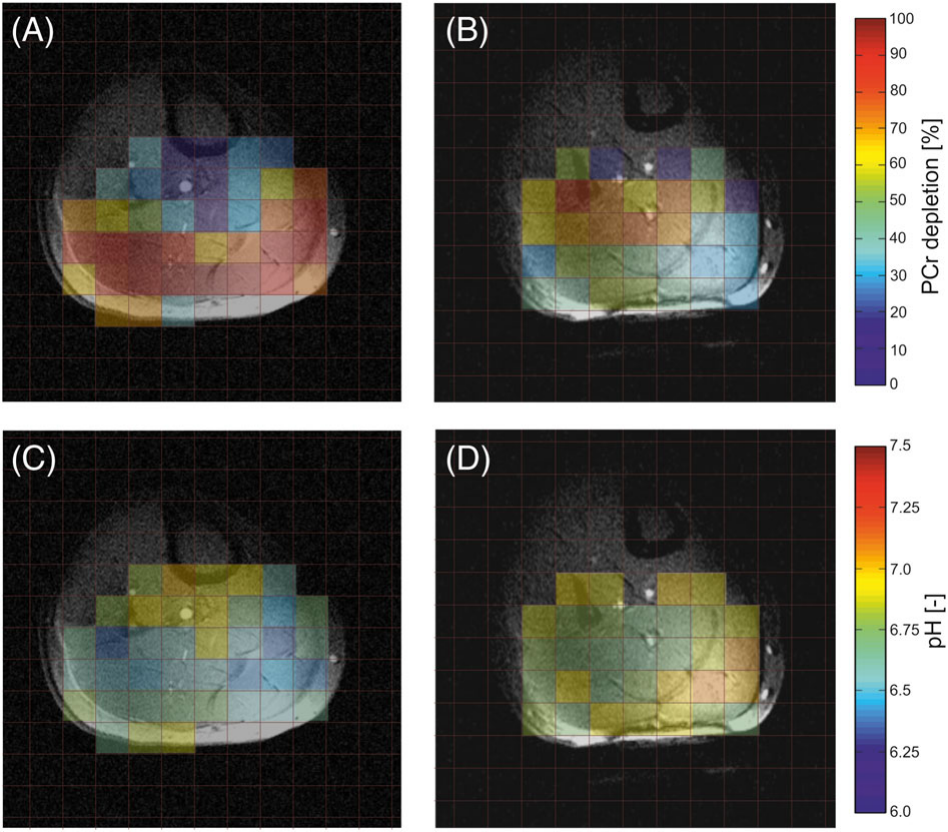
PCr depletion A,B and pH C,D maps of the same subject as a result of a plantar flexion exercise performed with a straight knee A,C and with the knee flexed by about 60° B,D. Note that in A,C most work is performed by the two superficially located gastrocnemii and the deeper situated SOL muscle is not much involved, as demonstrated by higher PCr depletions and lower pH values, whereas in B,D these roles are reversed

Figure 5 depicts PCr depletion maps measured at the end of the exercise period for both knee positions (0° versus ~60° flexion). A clear difference in the involvement of the investigated muscle groups can be observed. While in the first case (0° flexion) the level of PCr in the SOL muscle is almost unaffected, during the second exercise (~60° flexion) the SOL muscle is the one with the highest PCr depletion. The difference in exercise‐induced drop in PCr signal between the two dynamic experiments was found to be statistically significant (*p* < 0.05) for all three muscle groups (i.e. lower for both gastrocnemii and higher for SOL in the second exercise). Significantly shorter *τ*_PCr_ and higher Q_max_ were calculated for the SOL muscle in the second experiment; however, there were no statistical differences in these parameters found between the two experiments for either GM or GL. Table 4 summarizes the direct comparison of the measured and interpreted measures of mitochondrial function using these two exercise protocols.

**Table 4:** ^31^P–MR measures of mitochondrial function measured and derived from the dynamic examinations in five subjects with different knee angulations (0° and ~60°) (*n* = 5)

	GL		GM		SOL	
Knee flexion [°]	0	60	0	60	0	60
**PCr drop [%]**	75.4 ± 17.0	24.2 ± 12.7§	68.4 ± 24.9	20.4 ± 9.2§	18.0 ± 8.5	43.2 ± 19.1*
***τ***_**PCr**_ **[s]**	60.8 ± 10.1	57.7 ± 24.9	58.5 ± 21.7	58.7 ± 11.9	70.3 ± 12.0	42.5 ± 6.9§
**pH**_**end_exercise**_	6.76 ± 0.15	6.94 ± 0.07*	6.76 ± 0.20	7.00 ± 0.05*	6.93 ± 0.14	6.93 ± 0.14
***V***_**PCr**_ **[mM/s]**	0.47 ± 0.08	0.24 ± 0.13*	0.43 ± 0.18	0.21 ± 0.09*	0.12 ± 0.07	0.47 ± 0.27*
***Q***_**max**_ **[mM/s]**	0.60 ± 0.13	0.44 ± 0.18	0.59 ± 0.22	0.45 ± 0.15	0.33 ± 0.14	0.80 ± 0.34*

Data are given as mean ± standard deviation. Significant differences between exercise protocols are depicted as follows:

* *p* < 0.05 and

§ *p* < 0.01.

## 4 DISCUSSION

In our study, we propose dynamic spiral‐accelerated ^31^P–MRSI as an efficient method for simultaneous mapping of the temporal changes in PCr and intracellular pH during plantar flexion exercise of the calf muscle at 7 T. This method allows the assessment of muscle‐specific oxidative metabolism during and after exercise. The performance of our rapid spiral‐encoded ^31^P–MRSI method and its feasibility for dynamic experiments was investigated in phantom experiments and *in vivo* in a group of 12 healthy volunteers. Using this rapid technique we were also able to show the shift of the workload from the gastrocnemius to the SOL muscle resulting from different knee angulation during plantar flexion exercise. This is demonstrated by differences in the PCr drop between respective muscle groups in our two exercise set‐ups.

The conventional approach to dynamic ^31^P–MRS of using the sensitive volume of the RF coil for signal localization^16^, ^17^ restricts investigations to protocols with equal muscle involvement or to studies on superficial muscle groups only. Previous reports indicated that acquisition of signals from several muscles with different levels of recruitment risks biasing the results, as it does not reflect the real metabolic status of the individual muscles^27^, ^45^ and can affect the comparability of results between sites.^46^ Thus, several ^31^P–MR localization methods attempting to improve spatial specificity have recently emerged. On one hand, this includes ^31^P–MRS techniques, focused on single muscles.^24^, ^25^, ^45^ On the other hand, ^31^P–MRI techniques aim to map oxidative metabolism over the whole FOV simultaneously.^28^, ^29^, ^30^, ^31^, ^47^

Single‐voxel spectroscopy (SVS) provides an accurate localization of a cuboid volume as long as selective RF‐pulses with broad bandwidth are applied.^48^ However, SVS typically targets only a single muscle at a time. An interleaved technique providing information from two voxels within one dynamic experiment was suggested recently,^23^ but this still lacks the high spatial resolution of our MRSI approach. Furthermore, accurate spatial selection of SVS at 7 T requires relatively long *T*_E_, thus reducing sensitivity for important ^31^P metabolites such as ATP.^25^ The proposed MRSI approach is FID based, and therefore does not require such long *T*_E_.

^31^P–MRI techniques, recently proposed for mapping of muscle oxidative metabolism,^28^, ^29^, ^30^, ^31^, ^47^ sacrifice spectral selectivity in favor of spatial specificity. The disadvantage of mapping of single‐metabolite (i.e. PCr) resonances is that information about pH is lost. A simultaneous frequency‐selective ^31^P–MRI of PCr and P_i_ can utilize the phase maps of P_i_ to calculate pH.^31^ However, such pH measurements are slightly biased if the phase information is not derived from P_i_ only. Our sampling approach benefits from the excellent spectral resolution that is maintained thanks to the use of temporal interleaving, and thus pH can be accurately determined from the frequency difference between PCr and P_i_ 40, 42 in the same way as in conventional ^31^P–MRSI. Even the ATP signal is mapped simultaneously, which can serve as an important concentration reference to directly calculate *Q*_max_, and thus obtain an interpretation of mitochondrial capacity.

Earlier dynamic ^31^P–MRSI experiments were limited by slow Cartesian phase encoding and, therefore, required lengthy exercise protocols (e.g. gated ^31^P–MRSI with 16 min of exercise) and enabled only relatively low spatial resolution using an 8 × 8 matrix (10.5 mL nominal voxel size).^32^ On the other hand, the effective temporal resolution achieved with such protocols is equal to the *T*_R_ used, i.e. 2.1 s in the aforementioned study. Here we report an MRSI sequence with spiral‐trajectory encoding with a 14 × 14 matrix (6.1 mL nominal and 7.56 mL effective spatial resolution) and 10 s temporal resolution. Besides the increased encoding speed of our spiral‐MRSI, which supported a 23 times higher temporal resolution in comparison with the elliptical phase‐encoded MRSI, the spiral‐MRSI also led to an improved PSF (~30% smaller area at FWHM). Although the increased temporal resolution comes at the cost of SNR, after matching the acquisition times and correcting for the differences in PSF the SNRs achieved with the spiral‐encoded MRSI and with the elliptical‐encoded MRSI were comparable. The difference in PSF area measured in the phantom experiments is in good agreement with the results of Andronesi et al.,^35^ even though that the experimentally determined PSF for spiral encoding looks a little different from the simulations recently provided for ^13^C–MRSI.^49^ This was probably caused by the gridding process.

Comparing our spatial and temporal resolution with ^31^P–MRI studies, Schmid et al. proposed interleaved PCr/P_i_ mapping using a 3D spoiled gradient echo sequence with a 16 × 16 × 4 matrix (3 mL nominal spatial resolution) and 10 s temporal resolution.^31^ Parasoglou et al. suggested a PCr‐only frequency‐selective compressed sensing accelerated 3D turbo‐spin echo sequence with a 24 × 24 × 8 matrix (1.6 mL nominal spatial resolution) and 12 s temporal resolution.^30^ Greenman and Smithline reported 2D PCr imaging with a 20 × 20 matrix (5.6 mL nominal spatial resolution) and 6 s temporal resolution at 3 T.^28^ Our spectral‐spatial encoding approach via spiral spectroscopic imaging, therefore, merges the spectral specificity of ^31^P–MRS and spatial selectivity of ^31^P–MRI. In particular, our spiral‐MRSI sequence: (i) provides full signal intensity detection for all metabolites within the spectral bandwidth via direct acquisition of the FID; (ii) features the same high spectral resolution as conventional non‐localized ^31^P–MRS sequences; and (iii) provides accelerated spatial encoding similar to ^31^P–MRI for metabolic mapping.

Spectral bandwidth and temporal resolution can be balanced as required, while maintaining the full SNR per unit time (SNR/*t*). Theoretically, the use of rewinder gradients (i.e. gradients that are necessary to return to the center of *k* space) can reduce SNR/*t* efficiency. However, we have minimized this source of SNR loss by maximizing the number of spectral interleaves and eliminating the need for spatial interleaves. Thus, the necessary rewinder is very short, which restricts the SNR/*t* loss to well below 5%. Our phantom measurements also validate that the SNR/*t* values for elliptically phase‐encoded and spiral‐encoded MRSI were nearly identical. The entire image reconstruction is implemented online. Thus, spectra could be directly inspected/processed on the MR scanner.

The PCr depletions and *τ*_PCr_ values obtained for the different muscles in the scope of this study are in good agreement with those reported previously in healthy volunteers.^21^, ^22^, ^31^ The significantly lower PCr drop in the SOL muscle, in comparison with both gastrocnemii, during typical plantar flexion exercise with straightened knee points towards its low involvement in this type of exercise. Minimal muscle recruitment in the performed exercise, alongside with the substantial distance of SOL from the RF coil used, yielded less reliable *V*_PCr_ and *Q*_max_ values for this muscle. A trend towards lower *Q*_max_ in SOL for plantar flexion with a straight knee was also observed by Fiedler et al.^21^ This is also supported by our finding that once the recruitment of SOL was substantially increased through the flexion of the knee, as suggested by Price et al.^20^ and as confirmed by the significantly higher PCr depletion (43.2 ± 19.1% at ~60° flexion versus 18.0 ± 8.5% at 0°) in our study, the *Q*_max_ measured in SOL rose significantly. Although both GL and GM were recruited to a lesser extent and experienced significantly lower PCr depletion in the second experiment, *Q*_max_ was not found to be significantly different between the measurements for either of these muscle groups.

The use of a surface coil does introduce FA variability within the MRSI matrix. To overcome this, *B*_1_^+^‐insensitive adiabatic excitation could be used. However, slice‐selective adiabatic excitation pulses needed for 2D–MRSI would be extremely long and specific absorption rate (SAR) demanding, requiring prolonged *T*_R_. Another option would be to use non‐selective adiabatic excitation combined with slice‐selective adiabatic refocusing or inversion pulses. These approaches would lead to undesirably prolonged *T*_E_ and increased SAR, or towards doubled acquisition time.^50^ Therefore, none of these possibilities constitutes a good option for dynamic examinations. Alternatively, it is possible to compensate for the *B*_1_^+^ inhomogeneity of a surface coil by acquiring a map of the actual flip angle distribution, as was performed in this study using the method of Chmelík et al.^37^ The use of RF coils with a more homogeneous *B*_1_^+^ distribution^29^, ^51^ would also be beneficial.

The spiral spectroscopic imaging readout is demanding for the scanner gradient hardware. This will induce slow frequency drifts over time, as reported previously.^52^ This is not a severe limitation for dynamic ^31^P–MRSI with high temporal resolution, because accurate investigation of the creatine kinase metabolism does not rely on good frequency stability, but frequency drift could be a problem if this readout is combined with frequency selective pulses. This should be kept in mind when spiral spectroscopic imaging or other fast spectral‐spatial encoding methods are combined with saturation transfer techniques.^38^, ^53^, ^54^ The use of interleaved navigators was shown to be an excellent way of correcting frequency drifts for spiral spectroscopic imaging when combined with frequency‐selective MEGA‐editing pulses.^55^ With a recently suggested approach it might be possible to use interleaved ^1^H navigators also for ^31^P–MRSI on common MRI systems.^56^ The spiral MRSI readout proposed in our study could potentially lead to small artifacts when the changes in metabolite concentrations occurring during the dynamic protocol happen in between the acquisitions of individual temporal interleaves. These would originate from the largest signal in the spectrum, which is in our case PCr. However, we did not observe any during our test measurements *in vivo*, thus these theoretically present artifacts seem to be at or below the level of noise. Nevertheless, the selected bandwidth and number of temporal interleaves should ensure that, even if present, they would not overlap with any visible ^31^P resonances. Another potential source of artifacts relates to the narrow acquisition bandwidth used. Metabolite signals that are outside the acquisition bandwidth of the spiral MRSI sequence, but are excited by the excitation pulse, such as α‐ATP, could be aliased into the acquired spectra. However, these aliased signals would have only a fraction of the original signal amplitude, due to PSF blurring of signals from outside the spectral width,^57^ and therefore were not observed in our *in vivo* data.

We conclude that spiral‐encoded ^31^P–MRSI is a powerful tool for dynamic mapping of the muscle oxidative metabolism, including localized assessment of PCr concentration, pH and ATP reference concentration with a high temporal and spatial resolution. This not only allows the assessment of muscle‐specific mitochondrial capacity for all muscles within a single slice in physiological studies, but has also great potential for identifying small, i.e. early staged, localized pathologic changes in injuries, myopathies or PAD.
